# Reduced dopamine transporter availability in drug‐naive adult attention‐deficit/hyperactivity disorder

**DOI:** 10.1002/pcn5.177

**Published:** 2024-02-18

**Authors:** Shuntaro Itagaki, Takashi Ohnishi, Wataru Toda, Aya Sato, Junya Matsumoto, Hiroshi Ito, Shiro Ishii, Ryo Yamakuni, Itaru Miura, Hirooki Yabe

**Affiliations:** ^1^ Department of Neuropsychiatry Fukushima Medical University Fukushima Japan; ^2^ Medical Affairs Division Janssen Pharmaceutical K.K Tokyo Japan; ^3^ Department of Pathology of Mental Diseases, National Institute of Mental Health National Center of Neurology and Psychiatry Tokyo Japan; ^4^ Department of Radiology and Nuclear Medicine Fukushima Medical University Fukushima Japan; ^5^ Department of Mind & Brain Medicine Fukushima Medical University Fukushima Japan

**Keywords:** adult attention deficit hyperactivity disorder, developmental disorder, dopamine transporters, single photon emission computed tomography

## Abstract

**Aim:**

This study aimed to clarify the abnormalities in dopamine transporter (DAT) availability in drug‐naive adult patients with attention‐deficit/hyperactivity disorder (ADHD) and the relationship between ADHD symptoms and abnormalities in DAT availability.

**Methods:**

Single‐photon emission tomography (SPECT) was performed using iodine‐123‐β‐carbomethoxy‐3β‐(4‐iodophenyltropane) (I‐123 β CIT) as a tracer to measure in vivo DAT availability in 20 drug‐naive patients with ADHD [mean age ± standard deviation (SD)]: 25 ± 3.44 years; male:female = 11:9] and 20 age‐ and sex‐matched healthy controls (HCs) (mean age ± SD: 23.9 ± 2.27 years). Comparisons of DAT availability between HCs and adult patients with ADHD and the association between symptom severity and DAT availability within the ADHD group were analyzed using Statistical Parametric Mapping 12.

**Results:**

Drug‐naive adults with ADHD showed significantly reduced DAT availability in the bilateral nucleus accumbens compared with HCs. Correlation analyses revealed a negative correlation between the severity of inattentive symptoms in adult patients with ADHD and DAT availability in the bilateral heads of the caudate nucleus, indicating the association between severe inattentive symptoms and lower DAT availability in the caudate nucleus.

**Conclusion:**

In drug‐naive adult patients with ADHD, DAT availability was reduced in the nucleus accumbens, an important part of the reward system. This finding indicates the importance of the DAT in the reward system in the pathogenesis of ADHD. Inattentiveness was associated with DAT availability in the caudate nucleus, suggesting involvement of the cortico‐striato‐thalamo‐cortical circuit.

## Graphical abstract

Compared with 20 aged‐ and sex‐matched healthy controls, drug‐naive adults with ADHD showed significantly decreased DAT availability in the bilateral nucleus accumbens.

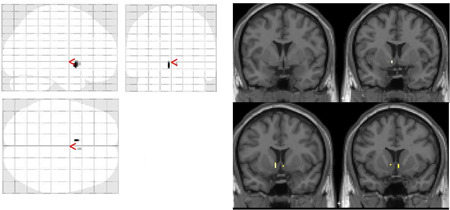

## INTRODUCTION

Attention‐deficit/hyperactivity disorder (ADHD) is a neurodevelopmental disorder characterized by symptoms of inattention, hyperactivity, and impulsivity, which affects millions of people worldwide. The estimated prevalence of ADHD among school‐aged children in the United States was 9.5% in 2007, and this has increased.^1^, ^2^ Although ADHD was previously thought to be a childhood disorder, since the 1970s, there have been increasing reports of ADHD persisting into adulthood from Europe and the United States.^3^ Subsequent follow‐up studies have shown that 49%–66% of those diagnosed with ADHD in childhood have ADHD that persists into adulthood, and the ADHD prevalence in adults was estimated to be 4.4% in the United States in 2006.^4^

ADHD is not simply a behavioral problem but is considered an innate brain dysfunction based on accumulated biological findings. ADHD is not only limited to its core symptoms but can also cause a variety of secondary and comorbid disorders as the child grows.^5^ Accordingly, there has been a shift in the classification categories for ADHD from attention‐deficit and disruptive behavior disorders in the *Diagnostic and Statistical Manual of Mental Disorders*, 4th edition, text revision^6^ (DSM‐IV TR) to neurodevelopmental disorders in the DSM‐5. Adult ADHD diagnosis requires confirmation of ADHD in childhood using pediatric records and caregiver interviews as well as current symptoms using questionnaires, such as the ADHD Report Scale (ADHD‐RS) and the Adult ADHD Self‐Report Scale (ASRS).^7^, ^8^ However, the development of biomarkers to assist in the diagnosis is necessary as some cases lack diagnostic evidence or are difficult to identify.

Dopamine transporter (DAT) plays a crucial role in regulating dopamine levels in the brain and has been implicated in the pathophysiology of ADHD.^9^ DAT knockout mice exhibit altered decision‐making processes and motivational states as well as motor and oral stereotypies,^10^, ^11^ which are closely related to the pathophysiology of ADHD. Therefore, in vivo measurement of DAT availability is a promising diagnostic biomarker. However, previous in vivo studies measuring DAT availability using positron emission tomography (PET) and single‐photon emission tomography (SPECT) in patients with ADHD have yielded inconsistent results.^12^, ^13^, ^14^ One potential explanation for these inconsistencies is the use of medications in patients with ADHD. ADHD medications, such as methylphenidate (MPH) and amphetamine, are known to affect DAT availability by blocking dopamine reuptake.^12^, ^15^ Wang et al. showed higher DAT availability in participants with ADHD than in controls after long‐term stimulant treatment.^16^ This raises the question of whether the differences in DAT availability observed in patients with ADHD on medication are due to the disorder itself or the effects of medication. Another problem is the establishment of regions of interest (ROIs). The basal ganglia consist of the putamen and caudate nucleus (the striatum), globus pallidus, substantia nigra, subthalamic nucleus, nucleus accumbens, ventral pallidum, and ventral tegmental area, each of which has a different function.^17^ However, some previous studies have examined the striatum as a single ROI, and the ROI varied among studies.^12^ This may also have contributed to inconsistencies in the results when evaluating DAT availability.

Therefore, this study aimed to address the above‐mentioned issues by examining the availability of DAT in drug‐naive adults with ADHD compared with healthy controls (HCs) using SPECT with iodine‐123‐β‐carbomethoxy‐3β‐(4‐iodophenyltropane) (I‐123butter CIT) as a tracer. We hypothesized that DAT availability in the basal ganglia, which is related to the reward system, would be lower in drug‐naive adults with ADHD than in the controls. A key feature of our study is the focus on drug‐naive (not only MPH but also other drugs for psychiatric diseases, including antidepressants) adults with ADHD. In adults with ADHD, comorbid disorders, such as mood disorders,^18^ often require treatments (such as antidepressants) that affect DAT availability.^19^ Consideration of these factors is expected to provide a more comprehensive understanding of the relationship between DAT and ADHD. Moreover, we conducted voxel‐by‐voxel analyses to avoid bias due to the setting of ROIs in the basal ganglia.

## METHODS

### Participants

Patients with adult ADHD were recruited from the outpatient psychiatry unit of Fukushima Medical University and neighboring psychiatric hospitals between February 1, 2017 and June 1, 2022. After explaining the study purpose, potential participants were referred to the outpatient psychiatry unit of our center. The HCs were recruited through local advertisements at Fukushima Medical University. This study included 20 treatment‐naive adult patients with ADHD (male:female = 11:9) and 20 age‐ and sex‐matched HCs.

At least two qualified psychiatrists diagnosed the patients using the criteria outlined in the DSM‐5. Participants with comorbid psychiatric disorders other than ADHD were excluded using a structured clinical interview based on the DSM‐5, clinical version. Participants with neurological or medical conditions that could potentially affect the central nervous system, such as atypical headaches, history of head trauma with loss of consciousness, thyroid disease, epilepsy, seizures, substance‐related disorders, or mental retardation, were also excluded. Participants with a history of illicit drug use, antidepressants (at least not over the last 3 months), or other psychoactive medications were excluded.

The presence and severity of ADHD symptoms in both patients with ADHD and HCs were assessed using Conners’ Adult Attention Deficit Hyperactivity Disorder Rating Scale (CAARS). Intellectual performance was evaluated using the Japanese version of the Wechsler Adult Intelligence Scale III (WAIS‐III). The WAIS provides a standardized full‐scale intelligence quotient (IQ) based on subtests that measure the levels of verbal [verbal IQ (VIQ)] and nonverbal knowledge and reasoning [performance IQ (PIQ)]. Written informed consent was obtained from all participants before enrollment. This study was approved by the Research Ethics Committee of Fukushima Medical University (approval code: No. 2693; approval date: April 13, 2016) and conducted in accordance with the Declaration of Helsinki.

### Measures

#### SPECT acquisition

SPECT imaging was performed using a triple‐headed rotating gamma camera (Canon GCA‐9300R, Version 2.2SP0001J; Canon Medical Systems, Otawara, Japan), which has a spatial resolution of 8.5 mm with a fan beam high‐resolution collimator (FANHR). I‐123 FP‐CIT (167 MBq) was administered intravenously as a bolus, and SPECT was performed 3 h after administration. Data were collected for 28 min for each patient. Two radiologists checked the images to ensure that they were of adequate quality and did not have any artefacts arising from head movement. SPECT acquisition was performed using the following parameters: matrix, 128 × 128; slice thickness, 1.72 mm; field of view, 220.16 mm; reconstruction method, ordered subset expectation maximization (iteration 4 and subset 15); Butterworth filter, 0.61 cycle/mm; and three‐point smoothing. Attenuation and scatter correction were not performed.

In the initial phase of image preparation, a processing console for the GCA‐9300R was used to generate the axial views of the scans. Subsequently, all SPECT data were imported into the DaTView software (Nihon Medi‐Physics Co., Ltd.), and ROIs were drawn based on the two‐box method introduced by Tossici‐Bolt et al. to calculate the specific binding ratio (SBR), asymmetry index, calibrated SBR, Z‐score, and calibrated asymmetry index.^20^

#### Magnetic resonance imaging acquisition

Whole‐brain data were acquired using a 3‐T SIEMENS Biograph mMR scanner. Foam padding was used to restrict head motion during acquisition. There were no effects of body movement. All acquisition protocols included a standard T1‐weighted magnetization‐prepared rapid gradient‐echo sequence (TI/TR = 800/1800 ms, TE = 1.98 ms, FA = 9, and X, Y, Z resolutions of 0.98, 0.98, 1 mm, respectively).

### Statistical analyses

As the sample size of this study was relatively small, the normal distribution and equal variances were evaluated using the Shapiro–Wilk and Levene tests, respectively. If these were not guaranteed, nonparametric tests, such as the Mann–Whitney *U* test and Spearman's rank–order correlation, were used.

#### Statistical analysis for demographic data

The chi‐squared test was used to analyze sex and handedness, and the independent two‐sample *t*‐test was used to compare age, IQ, and CARRS scores between patients with ADHD and HCs. Statistical analyses were performed using IBM SPSS Statistics Version 25 (IBM).

#### Voxel‐based statistical analysis

**Figure 1:**
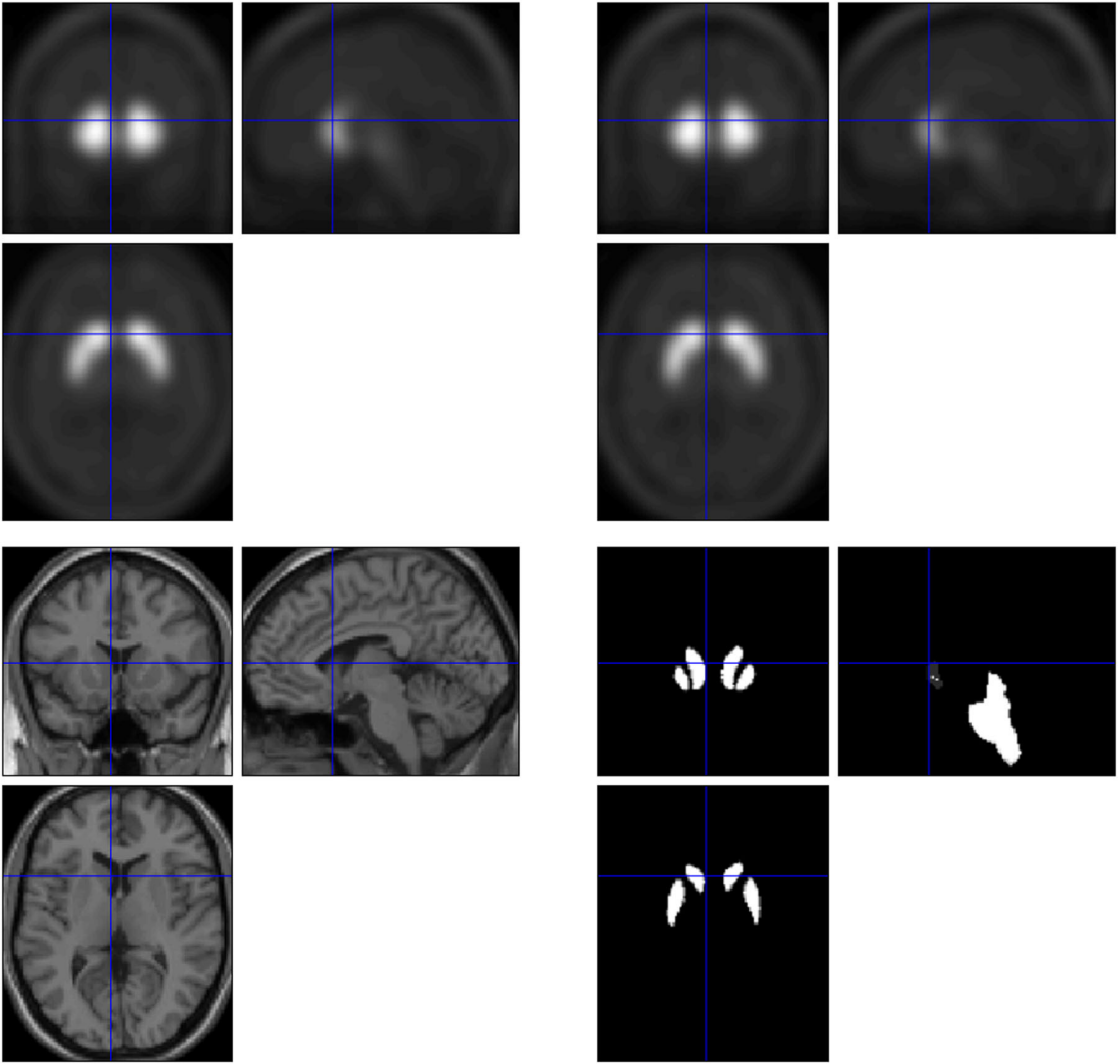
Top panels: mean dopamine transporter (DAT) images in each group after anatomical normalization. Upper left panel: mean DAT image for the healthy control (HC) group; upper right panel: mean DAT image for the attention‐deficit/hyperactivity disorder (ADHD) group. Lower right panel: voxel‐by‐voxel analysis performed on voxels within an anatomical mask.

Preprocessing of the DAT‐SPECT data was performed using Statistical Parametric Mapping 12 (SPM12) and entailed normalizing the data to an average‐sized DAT‐SPECT template. Data normalization to the Montreal Neurological Institute (MNI) space was performed using the “normalization” function. Subsequently, the images were warped non‐linearly from the MNI space to the native space of the T1‐weighted data using the spatial transformation parameters estimated for the gray matter (GM) and white matter (WM) probability maps. This allowed for the correction of partial volume effects using the modified Müller–Gartner method,^21^, ^22^ which is based on the convolution of DAT‐SPECT data with tissue classification maps estimated from T1‐weighted images. The GM SPECT images were normalized to the MNI space using parameters derived from the T1‐weighted data and scaled to the global mean GM signal for each patient. The top panels in Figure 1 depict the mean DAT image in each group after anatomical normalization (left: HC group, right: ADHD group). Finally, the SPECT images were smoothened using an isotropic Gaussian kernel with a full width at a half‐maximum of 6 mm.

To test the diagnostic efficacy, a two‐sample t‐test was applied to SPM12, and correlational analysis was performed. As the distribution of DAT is skewed toward the basal ganglia, midbrain, and so forth, voxel‐by‐voxel analysis was performed on voxels within an appropriate anatomical mask (Figure 1, lower right panel). A cluster threshold of *p* < 0.05 (uncorrected for multiple comparisons) was set for all voxel‐by‐voxel analyses. Since previous studies have provided evidence for abnormalities in DAT availability in the basal ganglia of patients with ADHD, we used a relatively lenient threshold of uncorrected *p* < 0.05.

#### Statistical analyses for volume of interest of significant clusters

Clusters that were significant in the SPM analyses were designated as volumes of interest (VOIs), and the average DAT availability values within the VOIs were extracted and tested. Owing to the relatively small sample size, these analyses were performed using nonparametric tests. The Mann–Whitney *U* test was used to evaluate the diagnostic effect on DAT availability. Spearman's rank correlation was computed to assess the relationship between ADHD symptom scores (CAARS *T* score of CAARS; DSM‐IV inattentive symptoms, DSM‐IV hyperactive/impulsiveness, and ADHD index) and DAT availability. Statistical analyses were conducted using R Statistical Software (Version 3.1.0; Foundation for Statistical Computing) and SAS Version 9.3 (SAS Institute Inc.).

## RESULTS

### Demographic data

The Shapiro–Wilk test indicated that the data were normally distributed at a significance level of 0.05. The Levene test indicated that the equality of error variances was assumed at a significance level of 0.05. Table 1 shows the demographic and clinical characteristics of adults with ADHD and HCs. All participants were Japanese who were unrelated to each other. Data obtained from 20 adult patients with ADHD (mean age: 25 years) and 20 HCs (mean age: 23.9 years) were analyzed (Table 1). Twelve patients had the inattentive type of ADHD, and eight patients had the combined type. Age, sex, or handedness did not differ significantly between patients with ADHD and HCs; however, patients with ADHD exhibited significantly lower IQ and higher CAARS scores than the HCs (Table 1).

**Table 1:** Demographic data of adults with ADHD and the healthy controls.

Demographic variables	Adults with ADHD	Healthy controls	*p* Value (two sample *t*‐test, *χ* ^2^ test)
Age, years [mean (SD)]	25.0 (3.44)	23.9 (2.27)	0.262
Sex (male/female)	11/9	11/9	1
Handedness (right/left‐handed)	18/2	19/1	0.548
Full IQ [mean (SD)]	108 (16.5)	122 (8.4)	0.002
Verbal IQ [mean (SD)]	110 (16.7)	123.6 (8.7)	0.003
Performance IQ [mean (SD)]	103.4 (16.1)	115.2 (11.1)	0.011
DSM‐IV inattentive symptoms [mean (SD)]	87.0 (4.5)	48.3 (6.6)	<0.001
DSM‐IV hyperactive/impulsive symptoms [mean (SD)]	69.4 (16.8)	49.5 (6.5)	<0.001
DSM‐IV total symptoms [mean (SD)]	82.6 (8.2)	48.7 (5.8)	<0.001
ADHD‐index [mean (SD)]	76.6 (8.3)	47.9 (5.6)	<0.001

Abbreviations: ADHD, attention‐deficit/hyperactivity disorder; DSM‐IV, *Diagnostic and Statistical Manual of Mental Disorders*, 4th edition, text revision; IQ, intelligence quotient; SD, standard deviation.

### Diagnostic effects on DAT availability

**Figure 2:**
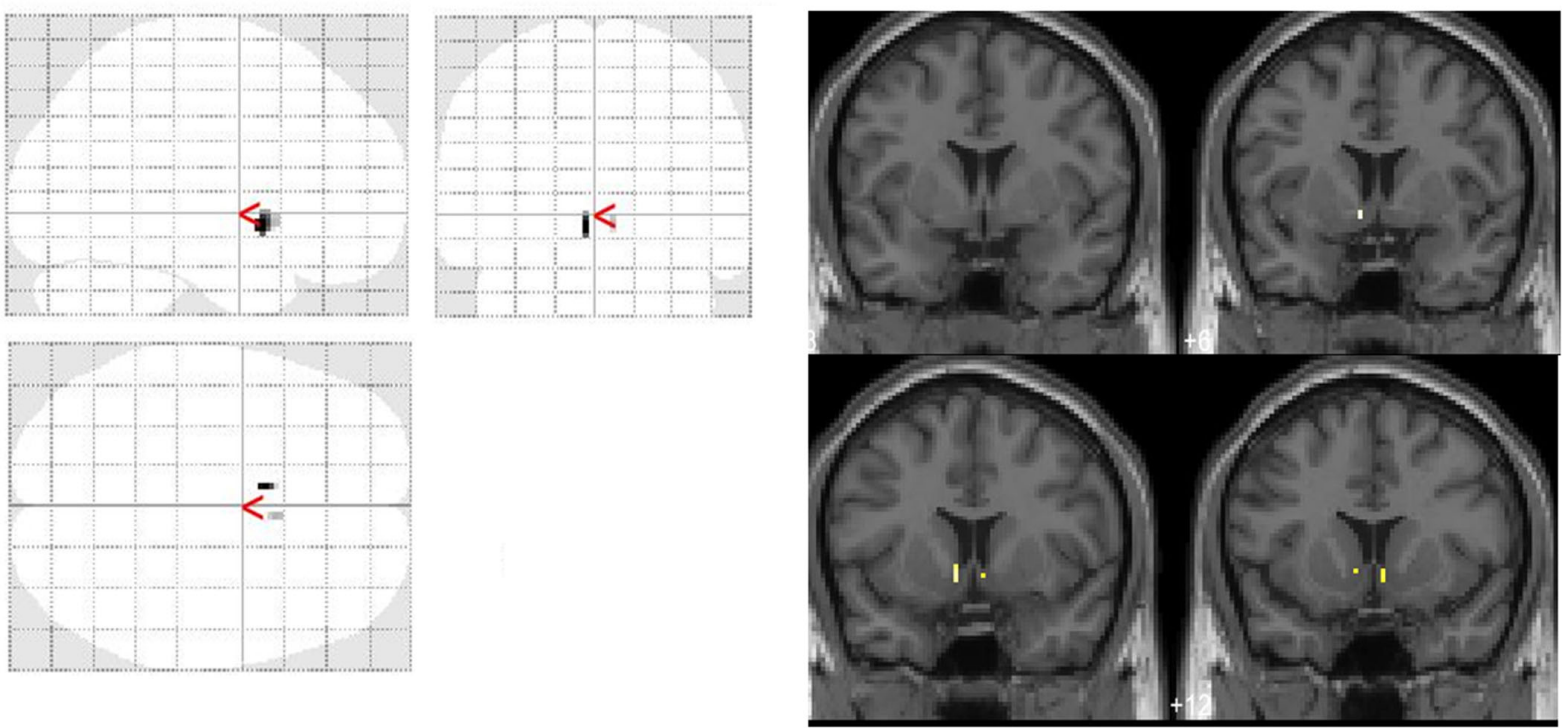
The left figure shows the Statistical Parametric Mapping (SPM) in the standard format as a maximum intensity projection viewed from the right, back, and top of the brain. Two significant clusters (*p* < 0.05, uncorrected) were observed in the left nucleus accumbens (peak Montreal Neurological Institute [MNI] coordinates: −6, 8, and −6) and right nucleus accumbens (peak MNI coordinates: 6, 12, and −6). The right figure shows significant clusters on coronal magnetic resonance imaging (MRI), showing a significant reduction in dopamine transporter (DAT) availability in the bilateral nucleus accumbens.

**Figure 3:**
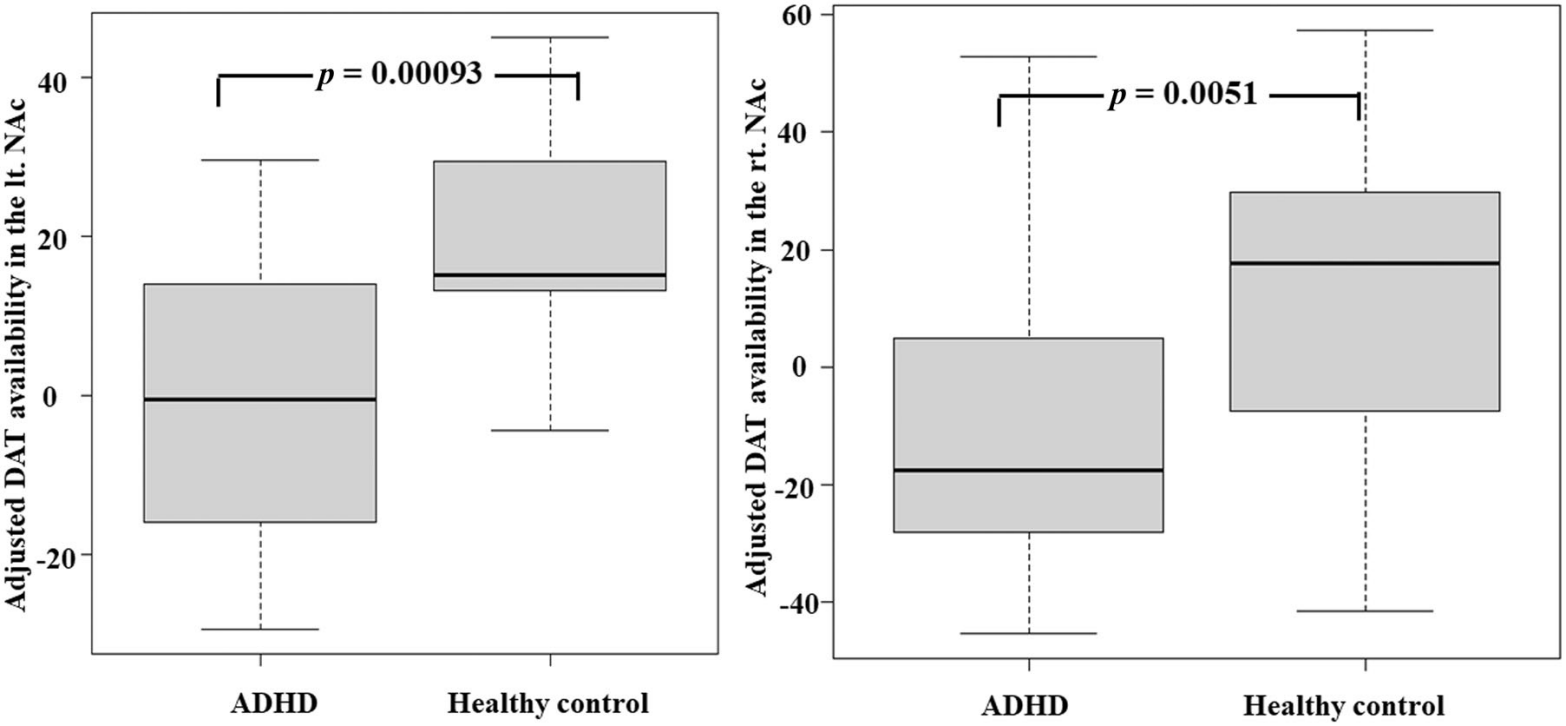
The left figure shows a box‐and‐whisker plot of mean dopamine transporter (DAT) availability within the cluster of the left nucleus accumbens (lt. NAc). The right figure shows a box‐and‐whisker plot of mean DAT availability within the cluster of the right nucleus accumbens (rt. NAc).

The voxel‐by‐voxel analysis revealed that DAT availability was decreased significantly in the bilateral nucleus accumbens HCs (Figure 2) and that DAT availability did not increase significantly in any region in adults with ADHD compared with HCs. VOI analysis revealed a significant decrease in DAT availability in the left (Mann–Whitney *U* test: *p* = 0.00093) and right nucleus accumbens (Mann–Whitney *U* test: *p* = 0.0051) in adults with ADHD compared with HCs (Figure 3). As there were significant differences in the IQ between adults with ADHD and HCs, an additional analysis of covariance with IQ as a covariate was performed, which revealed no significant interaction between the independent variable and covariate (left nucleus accumbens *p* = 0.0916, right nucleus accumbens: *p* = 0.39). We found a significant decrease in DAT availability in the left nucleus accumbens [*F*(1, 7.9615) = 1962.2, *p* = 0.007641] and a declining trend in the right nucleus accumbens [*F*(1, 3.9536) = 2623.8, *p* = 0.0542057) in adults with ADHD compared with HCs.

### Correlational analyses between DAT availability and symptomatic severity in adults with ADHD

**Figure 4:**
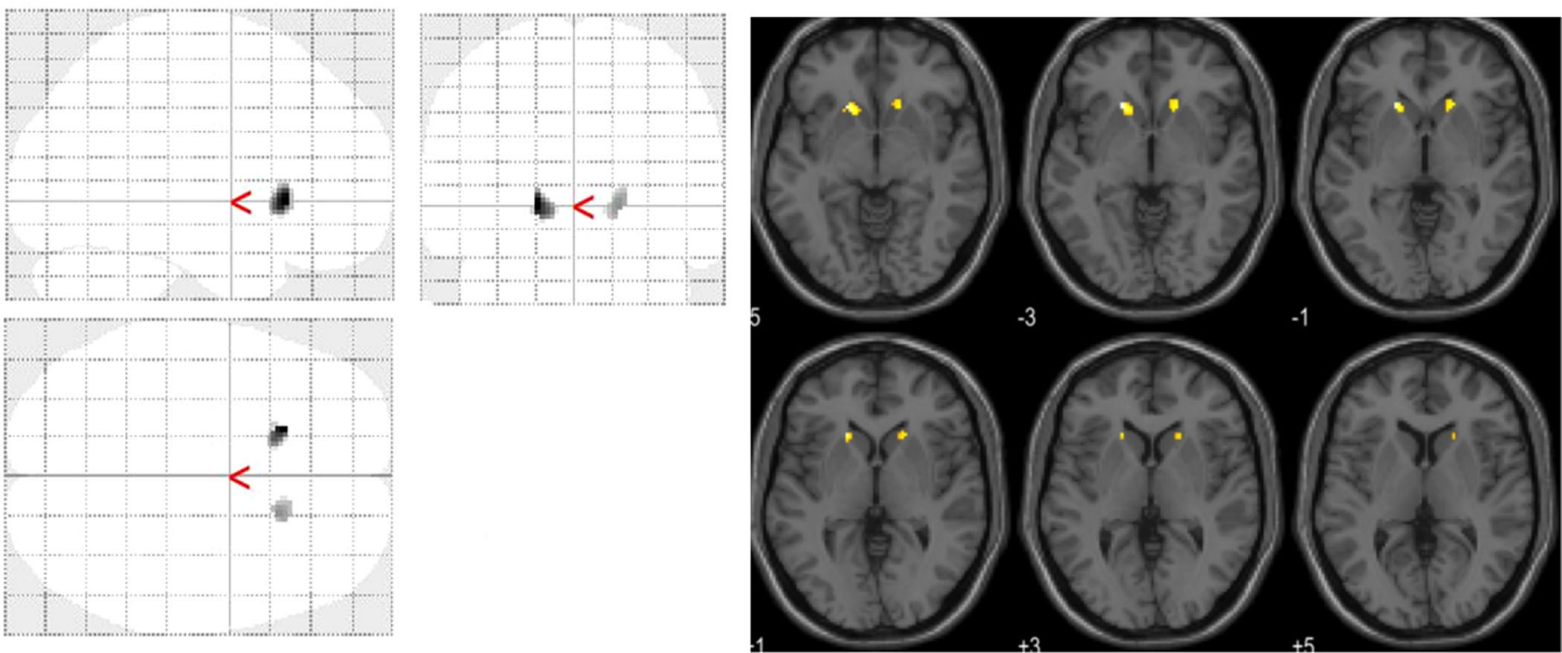
The left figure shows the Statistical Parametric Mapping (SPM) in the standard format as a maximum intensity projection viewed from the right, back, and top of the brain. Two significant clusters (*p* < 0.05, uncorrected) are seen in the left head of the caudate nucleus (peak Montreal Neurological Institute [MNI] coordinates; −18, 22, and −2) and right head of the caudate nucleus (peak MNI coordinates: 18, 20, and −2). The right figure shows significant clusters on axial magnetic resonance imaging (MRI), showing a significant negative correlation between the severity of inattentive symptoms of dopamine transporter (DAT) availability in the bilateral head of the caudate nucleus.

**Figure 5:**
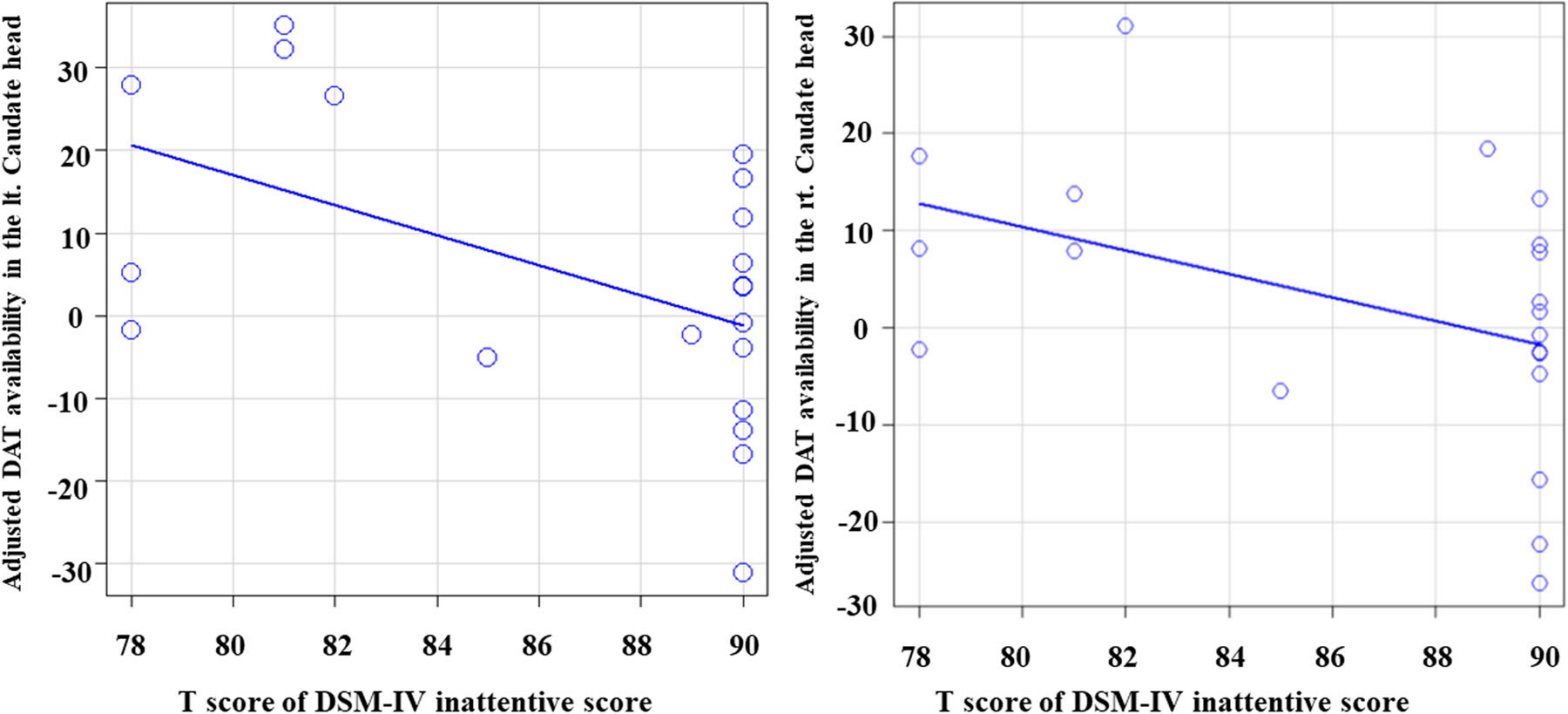
Scatter plots with the inattention severity score on the *x*‐axis and average dopamine transporter (DAT) availability within a cluster on the *y*‐axis. Left panel: Inattention severity scores and DAT availability in the left (lt.) caudate nucleus head shows a significant negative correlation (Spearman's rank correlation *r* = −0.43, *p* = 0.045). Right panel: The right (rt.) caudate nucleus head also shows a significant negative correlation (Spearman's rank correlation *r* = −0.48, *p* = 0.031) with inattentive severity scores.

Voxel‐by‐voxel correlation analysis only revealed a negative correlation between the DSM‐IV inattentive *T*‐score and DAT availability in the bilateral heads of the caudate nucleus (Figure 4). This indicated that more severe inattentive symptoms corresponded to lower DAT availability in the head of the caudate nucleus. No other significant correlations were observed. Figure 5 shows scatter plots with the inattentive severity score plotted on the *x*‐axis against the average DAT availability within a cluster on the *y*‐axis. The inattentive severity scores showed a significant negative correlation with DAT availability in the left (Spearman's rank correlation *r* = −0.43, *p* = 0.045; left panel of Figure 4) and right caudate nucleus heads (Spearman's rank correlation *r* = −0.48, *p* = 0.031; right panel of Figure 5).

## DISCUSSION

Our analysis with the two‐sample t‐test identified a decrease in DAT availability in the bilateral nucleus accumbens in drug‐naive adults with ADHD compared to that in HCs.

There was no significant decrease in DAT availability in the right nucleus accumbens in patients with ADHD after adjusting for IQ as a covariate, although a declining trend was observed. We believe that this observation may be attributed to the small sample size and the lack of statistical power associated with the reduction in the degrees of freedom arising from the addition of covariates. Therefore, we consider the results of the two statistical analyses to be essentially the same.

The analysis of covariance with IQ as a covariate showed that DAT availability in the right nucleus accumbens tended to decrease in patients with ADHD. This may be due to laterality in DAT availability in the nucleus accumbens. Studies have also reported that attentional control and intelligence are related to dopamine signals; therefore it is possible that the significant difference in the right nucleus accumbens disappeared due to adjustment.^23^ The most likely cause is the insufficient detection power. Therefore, further studies with a larger sample size may be necessary. This finding would essentially be a result of reduced DAT availability in the bilateral nucleus accumbens. Furthermore, we found a significant negative correlation between the severity of inattentive symptoms and DAT availability in the bilateral heads of the caudate nucleus in adults with ADHD.

Our results showing decreased DAT availability in the nucleus accumbens, which is the key brain region for reward and motivation,^24^ support the notion of impairment of the dopamine reward system in ADHD.^25^, ^26^ This abnormality in the dopamine reward system in ADHD^23^ is particularly observed in the mechanism of action of MPH,^26^, ^27^ one of the most effective treatments for ADHD that involves increasing striatal dopamine availability. Thus, this result demonstrates the importance of DAT in the pathogenesis of ADHD. However, in vivo measurements of DAT availability using PET and SPECT have yielded both high and low striatal DAT levels.^12^ A meta‐analysis suggested that this variability in DAT availability is associated with previous psychostimulant exposure, suggesting that DAT availability increases with psychostimulant administration.^12^ A longitudinal study measuring DAT availability before and after a long‐term MPH treatment in drug‐naive ADHD patients demonstrated increased DAT availability after long‐term MPH treatment, indicating that DAT adapts to synaptic dopamine levels and that chronic MPH treatment may result in upward regulation of DAT availability.^16^ In the present study of drug‐naive patients with ADHD, DAT availability decreased, supporting the hypothesis of a previous meta‐analysis.^12^

However, discrepancies exist in the results of studies that investigated drug‐naive patients, including the present study. Wang et al.^16^ reported an increase in DAT availability with MPH treatment, while reporting no difference in DAT availability in the control group in the untreated state. Similar to Wang et al., Volkow et al.^9^ reported reduced DAT availability in the left ventral caudate nucleus, nucleus accumbens, midbrain, and hypothalamus using C‐11 cocaine in untreated patients with ADHD compared with HCs. An I‐123 FP‐CIT SPECT study demonstrated reduced DAT availability in the striatum of drug‐naive adults with ADHD.^28^ The differences in the tracers and measurement methods (PET and SPECT) used in various studies may be responsible for these discrepancies; however, other factors need to be considered because discrepancies were noted even among studies using the same tracers and measurement methods.^9^, ^29^ We speculate that ADHD is a heterogeneous disorder that may have variations in DAT availability, which may be responsible for the differences in results among studies. This hypothesis indicates that the response to MPH treatment can be clinically predicted by measuring DAT, and is supported by a previous study showing that DAT availability is related to the response to MPH treatment.^29^

Since impulsive behavior is associated with abnormalities in the ventral striatal dopamine system (an important part of the reward and motivation system),^22^, ^29^, ^30^ we expected a correlation between DAT availability in the nucleus accumbens and the severity of hyperactive impulsive symptoms. However, there was no significant correlation between DAT availability in the nucleus accumbens and the hyperactivity–impulsivity scores. This negative finding may be attributed to the use of the CAARS to assess impulsive behavior and not reward sensitivity, as in the Behavioral Activation System (BAS) scale.^31^ Thus, we can only speculate that disturbances in the dopamine reward pathway may underlie the clinical evidence of abnormal reward responses in ADHD.

In contrast, a negative correlation was observed between the inattentiveness scores and DAT availability in the bilateral heads of the caudate nucleus, indicating that the decreased DAT availability in this region may be associated with increased inattentive symptoms in patients with ADHD. The caudate nucleus is a part of the cortico‐striato‐thalamo‐cortical circuit, which is involved in the regulation of attention and executive functions; dysfunction of this circuit is associated with ADHD symptoms.^32^, ^33^ In this context, our findings are consistent with the functional anatomy and pathophysiology of ADHD. Contrastingly, a previous study reported a significant correlation between inattentive symptom severity and DAT availability in the midbrain but not in the caudate nucleus.^9^ However, this study also observed an association between reduced dopamine D2/D3 receptor binding and the severity of inattentive symptoms.^9^ Thus, we speculate that altered dopaminergic function in the caudate nucleus plays an important role in the inattentive symptoms of ADHD.

This study had some limitations. First, the sample size was small, which may have limited the generalizability of the results. Additionally, the spatial resolution of SPECT is relatively lower than that of PET, which may affect the measurement accuracy. In the statistical analysis, an uncorrected and relatively lenient threshold of *p* < 0.05 was used, according to results of previous studies. Finally, the HCs in this study had high IQs.

In conclusion, this study provides further evidence of the involvement of DAT in the pathophysiology of ADHD. Decreased DAT availability in the nucleus accumbens suggests that interventions aimed at increasing DAT availability may be beneficial for treating the reward‐related symptoms of ADHD. The association between decreased DAT availability in the caudate nucleus and increased inattentive symptoms also suggests that interventions aimed at increasing DAT availability may be beneficial for treating inattentiveness. Further research with larger sample sizes and more rigorous statistical analyses are needed to confirm these findings and investigate the potential therapeutic implications.

## AUTHOR CONTRIBUTIONS

Shuntaro Itagaki contributed to data collection, data analysis, and drafting of the article. Takashi Ohnishi contributed to the research planning, data analysis, and writing of the article. Yamakuni Ryo contributed to the writing of this article. Wataru Toda, Aya Sato, and Junya Matsumoto supervised the survey. Hiroshi Ito, Shiro Ishii, Itaru Miura, and Hirooki Yabe contributed to planning of the investigation and supervision of the survey. All authors contributed to the interpretation of results, drafted the manuscript, participated in subsequent revisions, and read and approved the final version of the manuscript.

## CONFLICT OF INTERESTS STATEMENT

Dr Takashi Ohnishi is a full‐time employee of Janssen Pharmaceutical K.K. of Johnson and Johnson, Japan. Dr Hirooki Yabe is an Editorial Board member of *Psychiatry and Clinical Neurosciences Reports* and a co‐author of this article. To minimize bias, they were excluded from all editorial decision‐making processes related to the acceptance of this article for publication. The remaining authors declare no conflicts of interest.

## ETHICS APPROVAL STATEMENT

This study was approved by the Research Ethics Committee of Fukushima Medical University (approval code: No. 2693; approval date: April 13, 2016) and conducted in accordance with the Declaration of Helsinki.

## PATIENT CONSENT STATEMENT

All participants provided written informed consent for participation in this study.

## CLINICAL TRIAL REGISTRATION

UMIN000025183: The development of a diagnostic biomarker for adult attention‐deficit hyperactivity disorder: a multimodal approach.

## Data Availability

Owing to the nature of this research, the participants did not agree for their data to be shared publicly; therefore, supporting data are not available.
